# Ceramides and metabolic profiles of patients with acute coronary disease: a cross-sectional study

**DOI:** 10.3389/fphys.2023.1177765

**Published:** 2023-12-11

**Authors:** Liang Zhang, Dawei Tan, Yang Zhang, Yaodong Ding, Huiqing Liang, Gong Zhang, Zhijiang Xie, Nian Sun, Chunjing Wang, Bingxin Xiao, Hanzhong Zhang, Lin Li, Xiufeng Zhao, Yong Zeng

**Affiliations:** ^1^ Department of Cardiology, Beijing Anzhen Hospital, Beijing Institute of Heart, Lung and Blood Vessel Diseases, Capital Medical University, Beijing, China; ^2^ Heart Center, Beijing Chest Hospital, Capital Medical University, Beijing, China; ^3^ Department of Invasive Technology, Emergency General Hospital, Beijing, China; ^4^ Department of Cardiology, The First Affiliated Hospital of Hebei North University, Zhangjiakou, China; ^5^ Department of Cardiology, Beijing Daxing District People’s Hospital, Beijing, China; ^6^ Department of Cardiology, Handan First Hospital, Handan, China; ^7^ Beijing Health Biotechnology Co., Ltd., Beijing, China; ^8^ Beijing 21st Century International School, Beijing, China

**Keywords:** acute coronary syndrome, metabolic syndrome, ceramide, biomarker, stratification treatment, LC-MS/MS

## Abstract

Metabolic Syndrome (MS) is a rapidly growing medical problem worldwide and is characterized by a cluster of age-related metabolic risk factors. The presence of MS increases the likelihood of developing atherosclerosis and significantly raises the morbidity/mortality rate of acute coronary syndrome (ACS) patients. Early detection of MS is crucial, and biomarkers, particularly blood-based, play a vital role in this process. This cross-sectional study focused on the investigation of certain plasma ceramides (Cer14:0, Cer16:0, Cer18:0, Cer20:0, Cer22:0, and Cer24:1) as potential blood biomarkers for MS due to their previously documented dysregulated function in MS patients. A total of 695 ACS patients were enrolled, with 286 diagnosed with MS (ACS-MS) and 409 without MS (ACS-nonMS) serving as the control group. Plasma ceramide concentrations were measured by LC-MS/MS assay and analyzed through various statistical methods. The results revealed that Cer18:0, Cer20:0, Cer22:0, and Cer24:1 were significantly correlated with the presence of MS risk factors. Upon further examination, Cer18:0 emerged as a promising biomarker for early MS detection and risk stratification, as its plasma concentration showed a significant sensitivity to minor changes in MS risk status in participants. This cross-sectional observational study was a secondary analysis of a multicenter prospective observational cohort study (Chinese Clinical Trial Registry, https://www.who.int/clinical-trials-registry-platform/network/primary-registries/chinese-clinical-trial-registry-(chictr), ChiCTR-2200056697), conducted from April 2021 to August 2022.

## 1 Introduction

Acute coronary syndrome (ACS) is a diverse and complicated medical condition that remains a leading cause of morbidity and mortality worldwide (Ma et al., 2020; Bergmark et al., 2022), despite the provision of optimal medical care. Studies (Sinha et al., 2016; Younis et al., 2016) have shown that over half of ACS patients suffer from metabolic complications, leading to an increased cardiovascular death rate and long-term adverse events. However, due to the complex and varying nature of patients, designing an effective metabolic risk stratification for ACS remains a challenging task.

Metabolic Syndrome (MS) is a group of age-related metabolic conditions characterized by symptoms such as abdominal obesity, dyslipidemia, hypertension, and insulin resistance (IR), also known as MS risk factors. The prevalence of MS has experienced a rapid increase on a global scale, with a significant increase in the US from 32.5% in 2012 to 36.9% in 2016. In China, the estimated prevalence rate reached 11%, according to the Expert Consensus on Drug Treatment for Metabolic Syndrome in the Elderly in China (2022) (Saklayen, 2018; Hirode and Wong, 2020; Expert Consensus on Drug Therapy of Metabolic S yndrome in the Elderly in China2022Writing Group, 2022). In patients with ASCVD, the prevalence of MS is greater than 50%, and MS is associated with a >2-fold increased risk for ASCVD and cardiovascular mortality (Sperling et al., 2015)**.** The presence of MS promotes the development of atherosclerosis and significantly increases the probability of morbidity and mortality in ACS patients (Sato, 2014). A large-scale, multi-ethnic study conducted by Anand and the INTERHEART Investigators (Mente et al., 2010) found that the risk of MS on myocardial infarction was significantly stronger than many other risk factors. Therefore, having a precise understanding of a patient’s MS status ahead of time presents a significant advantage for planning individualized and effective treatment strategies, and enables a more cost- and time-effective medical system. Previous findings (Aguilar-Salinas et al., 2005; Grundy, 2008) have highlighted the limitations of using monotherapeutic MS diagnosis to assess MS risks and the potential to misidentify high-risk subjects. As a result, there is a need for the development of novel and precise biomarkers to help medical staff screen patients’ cardiometabolic conditions and achieve a MS risk stratification system.

**MS** is associated with a range of biomarkers, including adiponectin, leptin, the leptin/adiponectin ratio, TNF-α, interleukin-6, interleukin-10, PTX3, ghrelin, uric acid, and OxLDL (Falahi et al., 2015; O’Neill et al., 2016; Rafaqat et al., 2021). These biomarkers have been shown to be disrupted in MS patients compared to healthy controls and can be measured using blood tests. However, the diagnostic process can be complicated by the need for additional tests, such as glucose tolerance and waist circumference, as well as low-yield and laborious analytical methods, such as ultracentrifugation and density-based assays, or the use of expensive commercial kits, which highlights the need for a simple, blood-based test that can provide a minimally invasive, sensitive, and specific diagnosis for MS.

Ceramides, a group of biologically active lipids produced through metabolism, have been identified as potential modulators for various diseases, including Alzheimer’s disease, coronary artery disease, and multiple sclerosis (Bismuth et al., 2007). These lipids have been found to exert anti-diabetic, cardioprotective, and insulin-sensitizing effects when their levels are reduced (Holland et al., 2011). Elevated levels of ceramides have been observed in several conditions, including obesity, diabetes, hypertension, heart failure, and atherosclerosis, leading to ongoing research into developing therapeutic drugs targeting ceramides (Bismuth et al., 2007). Ceramide dysregulation is also identified in a range of MS cases (Yang et al., 2009; Green et al., 2021; Magnan and Stunff, 2021). The history of ceramide study as potential biomarkers for cardiovascular diseases can be traced back to as late as 70s and carries on till now while certain plasma ceramides (Cer16:0, Cer18:0, and Cer24:1) and ceramide ratios (Cer16:0/Cer24:0, Cer18:0/Cer24:0, and Cer24:1/Cer24:0) have been found repeatedly to display independent predictive value for cardiovascular events (Laaksonen et al., 2016a; Meeusen et al., 2018; Tu et al., 2020). Recent lipidomic studies have also revealed the potential predictive value of ceramides, such as Cer17:0, Cer20:0, and Cer24:1, for adverse cardiovascular events among cardiovascular patients with multiple metabolic syndrome risk factors (Huynh et al., 2019).

Composed of a sphingosine base and a fatty acid tail, ceramide molecules have molecular weights ranging from some hundred to around 1,000 Da, making it feasible **for** tandem mass spectrometry analysis, that is, often coupled with liquid chromatography separation technique (LC-MS/MS). Method-wisely speaking, it provides a significant advantage in blood-based ceramide analysis compared to other conventional biomarkers, as modern mass spectrometry assays offer high-resolution, high-throughput, and highly specific quantification procedures, enabling accurate measurement at the molecular level.

In this study, our objective was to examine the association between specific ceramides and MS risk factors in patients with acute coronary syndrome (ACS) and to determine if ceramides can be used as a substitute for metabolic profiling in this population. Additionally, we aimed to explore the feasibility of developing a comprehensive medical system that utilizes individual plasma **ceramide** levels to differentiate MS patients based on their level of severity, particularly in ACS cases. Based on literature review results, the ceramides selected for analysis were Cer14:0, Cer16:0, Cer18:0, Cer20:0, Cer22:0, and Cer24:1. By combining LC-MS/MS assay with statistical analysis, our goal was to provide a new and innovative way for clinical staff to assess and interpret plasma ceramides as potential biomarkers for ACS patients.

## 2 Materials and methods

### 2.1 Ethics approval of the research protocol

This study was approved by the ethics committee of the Beijing Anzhen Hospital, Capital Medical University. All patients or their legal proxies gave written informed consent.

### 2.2 Study design and participants

**FIGURE 1:**
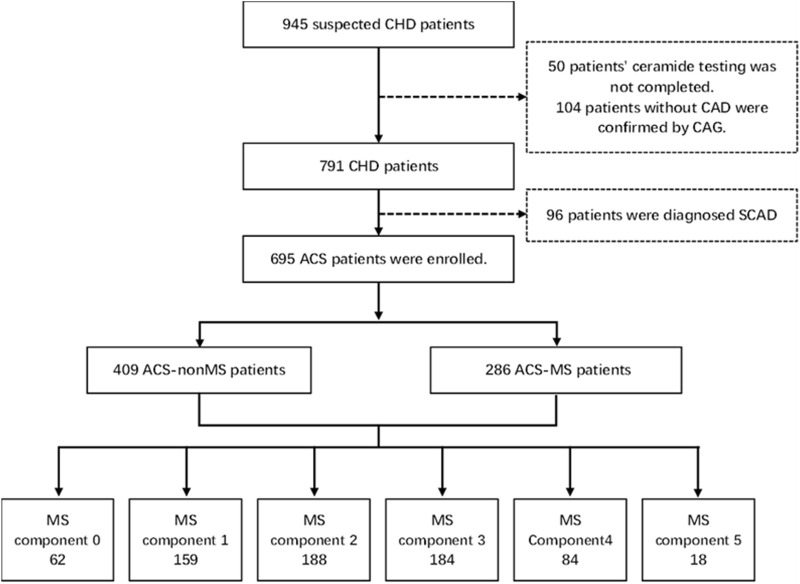
Study frame diagram. CAD, coronary artery disease; CAG, coronary artery angiography; SCAD, stable coronary artery disease; ACS, acute coronary syndrome; MS, metabolic syndrome.

This cross-sectional observational study was a secondary analysis of a multicenter prospective observational cohort study (Chinese Clinical Trial Registry, ChiCTR-2200056697), conducted from April 2021 to August 2022. A total of 945 patients undergone coronary **angiographs** were included initially (Chinese Clinical Trial Registry, ChiCTR-2200056697). The inclusion criteria were: 1) patients aged 18 years or older and diagnosed as having ACS according to the ACS diagnostic criteria and 2) patients had a complete clinical data record. The exclusion criteria were pregnant women, familial hypercholesterolemia patients, patients suffering from bleeding disorders, patients with neoplasms with a life expectancy <1 year, mental illness, drug abuse or alcohol dependence, chronic kidney disease (estimated glomerular filtration rate <60 mL/min/1.73 m^2^) and patients with a clear non-cardiac chest pain. Another 50 patients were excluded as there was **no** adequate blood specimen and 104 patients were excluded due to their insignificant coronary stenosis (<50%). After excluding another 96 patients with stable coronary artery disease, 695 participants with ACS were enrolled in the study cross-sectionally. Among these, there were 286 patients with metabolic syndrome (ACS-MS), and the other 409 patients with only ACS (ACS-nonMS) as the control group (Figure 1).

### 2.3 Data collection

Patients’ demographic and clinical characteristics were obtained by reviewing their medical records. Reported concentrations of fasting blood glucose (FBG), glycated hemoglobin A1c (HbA1c), uric acid (UA), blood lipids, and C-reactive protein (CRP) were measured by the standard laboratory techniques (Roche Diagnostics, Mannheim, Germany). In addition, traditional cardiovascular risk factors such as hypertension (systolic blood pressure (SBP) ≥ 140 mmHg, diastolic blood pressure (DBP) ≥ 90 mmHg, or taking antihypertensive medication), T2DM (FBG ≥7.0 mmol/L, HbA1c ≥ 6.5%, or taking antihyperglycemic medication) were also analyzed.

### 2.4 Definitions of MS and MS risk factor components

MS is defined according to the 2020 Chinese Diabetes Society criteria (Zhu, 2021). Patients with at least 3 of the following criteria are considered subjected to MS: 1) central obesity: waist circumference (WC) of ≥90 cm in men and ≥85 cm in women. 2) Hypertriglyceridemia (≥1.7 mmol/L), 3) low HDL-C (<1.04 mmol/L), 4) hypertension and 5) Type 2 diabetes. The five criteria are also termed risk factors for the unity of this paper. We utilized the BMI ≥28 kg/m^2^ as a diagnostic criterion of central obesity proposed by the Working Group on Obesity of China (WGOC) (Zhou, 2002), which had been adopted and verified in previous studies (Samanta et al., 2020).

### 2.5 Quantification of ceramides

Fasting blood samples were pre-treated with EDTA anticoagulation prior to the laboratory analysis. Samples were stored at 4 C for less than 1 h after collection for fast analysis and then frozen at −80°C for short-term storage until analysis for no more than 2 weeks period. LC–MS/MS system (AB Sciex Triple Quad™ 4500MD (Sciex, Framingham, MA, United States)) was used to simultaneously quantify the circulating plasma ceramides (Cer14:0, Cer16:0, Cer18:0, Cer20:0, Cer22:0, and Cer24:1) as described in our recently published paper^30^. In short, protein precipitation with deuterated internal standards was applied for sample preparation. Chromatographic separation based on C8 column combined with multiple reaction monitoring (MRM) mode under positive electrospray ionization (ESI) was utilized, in which only the selected precursor and product ions with a defined mass/charge (m/z) ratio were identified, thus ensuring the quantification of the target analytes.

### 2.6 Statistical analyses

All statistical analysis was conducted by SPSS 22.0 (IBM, Inc., Chicago United States). *p* < 0.05 was regarded as statistically significant. To describe the demographic and baseline characteristics of cardiovascular disease patients, descriptive statistics (mean, standard deviation) were calculated for all study variables by cross-sectional status. A Shapiro-Wilk test for normal distribution was performed. Continuous variables were expressed as the mean ± SD, and categorical variables were expressed as a n (%). **Intergroup** comparison was evaluated by a Chi-squared test or Z-test. We assumed that circulating **ceramide** levels increased progressively with increasing numbers of MS risk factors and divided the study population into subgroups according to the number of MS risk factors: 0 (M0), M1, M2, M3, M4, and M5. The study population **was** further re-categorized into three groups based on the MS risk factor combination. For example, **subgroups** with 0 and 1 MS **risk factors** were re-grouped as M0-1, 2, and 3 MS **risk factors** as M2-3, 4, and 5 MS risk factors as M4-5. The differences in plasma **ceramide** levels between groups were assessed with ANOVA test. In addition, Spearman’s correlation analysis was performed to assess the relationship between individual ceramides and traditional cardiovascular risk factors. Logistic regression analysis was used to investigate the relationship between the ceramides and MS risk factors. Risk factors with a *p* < 0.20 from the univariate analyses were included in the multivariate analysis to assess the independent effect of ceramides. ORs and 95% confidence intervals (CIs) were calculated.

## 3 Results

### 3.1 Baseline characteristics of the study population

**FIGURE 2:**
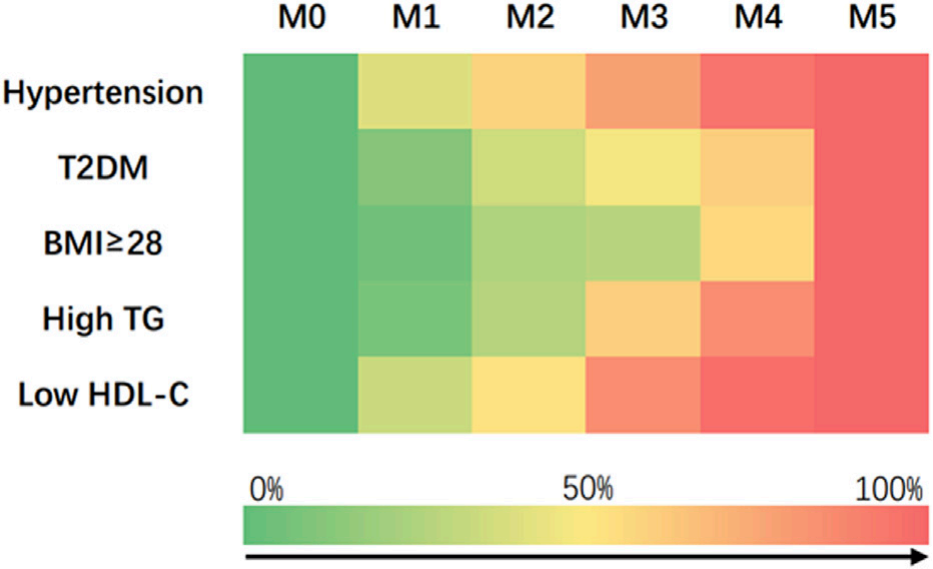
Heat map showing the prevalence of cardiometabolic risk factors in M0 (*n* = 62), M1 (*n* = 159), M2 (*n* = 188), M3 (*n* = 184), M4 (*n* = 84) and M5 (*n* = 18).

A total of 695 patients diagnosed with ACS at Beijing Anzhen Hospital participated in this study. The demographic and clinical characteristics of the population were summarized in Table 1. The average age was 59.2 ± 9.4 among ACS-MS participants and 60.2 ± 10.3 among ACS-nonMS participants. There were **significant** statistical differences within ACS-MS group with respect to the number of MS risk factors. The prevalence of hypertension and T2DM was higher in ACS-MS group than in ACS-nonMS group, while higher plasma levels of glucose, uric acid, LDL-C, and TG and lower levels of eGFR and HDL-C were found to be associated with ACS-MS group. The proportion of patients with unstable angina was not statistically different between ACS-MS and ACS-nonMS groups. Differential prevalence of metabolic disorder in patients with MS was presented by Heat map, and the frequency of conventional cardiovascular risk factors gradually increased with the accumulated number of MS risk factors. (Figure 2 **)**

**TABLE 1:** Clinical characteristics of the subjects categorized by disease status.

Characteristic	ACS-MS group (n = 286)	ACS-nonMS group (n = 409)	*p*-value
Demographic			
Age (years)	59.22 ± 9.41	60.15 ± 10.28	0.225
Male n (%)	219 (76.6%)	295 (72.1%)	0.189
BMI (kg/㎡)	27.24 ± 3.25	24.92 ± 3.07	0.000
Unstable angina n (%)	265 (92.7%)	382 (93.4%)	0.705
Hypertension n (%)	242 (84.6%)	176 (43.0%)	0.000
T2DM n (%)	159 (55.6%)	83 (20.3%)	0.000
Smoking n (%)	131 (45.8%)	177 (43.3%)	0.509
Laboratory value			
TG (mmol/L)	2.10 (1.59–2.88)	1.19 (0.92–1.54)	0.000
TC (mmol/L)	3.95 ± 1.02	4.02 ± 1.12	0.397
HDL-C (mmol/L)	0.91 ± 0.17	1.17 ± 0.26	0.000
LDL-C (mmol/L)	2.10 ± 0.78	2.21 ± 0.96	0.114
SdLDL-C (mmol/L)	0.82 ± 0.42	0.66 ± 0.56	0.000
FFA (mmol/L)	0.49 ± 0.46	0.56 ± 0.55	0.074
Hs-CRP (mg/dL)	1.20 (0.66–2.60)	0.92 (0.53–1.98)	0.001
FBG (mmol/L)	6.67 (5.47–8.49)	5.32 (4.81–6.39)	0.000
HbA1c (%)	6.40 (5.80–7.40)	5.80 (5.50–6.30)	0.000
eGFR (mL/min/1.73m^2^)	90.36 ± 17.54	93.46 ± 15.23	0.015
HCY (umol/L)	14.10 (12.30–17.10)	13.60 (11.90–15.90)	0.020
UA (umol/L)	348.30 ± 93.20	322.63 ± 94.55	0.000
Cermaides (umol/L)			
Cer14:0	2.84 ± 1.38	2.87 ± 1.45	0.779
Cer16:0	172.25 ± 50.72	163.11 ± 51.43	0.020
Cer18:0	48.12 ± 19.83	42.78 ± 18.49	0.000
Cer20:0	61.47 ± 21.53	54.93 ± 21.62	0.000
Cer22:0	471.04 ± 178.10	412.60 ± 153.88	0.000
Cer24:1	501.36 ± 200.73	456.94 ± 176.86	0.003

Values are reported as n (%), mean ± SD, or median (interquartile range).

BMI, body mass index; SdLDL-C, Small dense low-density lipoprotein cholesterol; TC, total cholesterol; TG, triglyceride; HDL-C, high-density lipoprotein cholesterol; LDL-C, low-density lipoprotein cholesterol; FBG, fasting blood glucose; HbA1c, glycated hemoglobin A1c; Hs-CRP, High sensitivity C-reactive protein; eGFR, estimated glomerular filtration rate; HCY, homocysteine; UA, uric acid.

### 3.2 Correlation of ceramides and traditional cardiovascular risk factors

The Pearson correlation coefficients for the levels of individual ceramides and those for traditional cardiovascular risk factors for the whole study population were calculated (Table 2). The results demonstrated that the levels of ceramides and their ratios had a mild-to-moderate correlation with those of traditional cardiovascular risk factors. Off note was the relatively strong correlation of ceramides with the levels of blood lipids including TC, TG, HDL-C, LDL-C, SdLDL-C, **and** FFA.

**TABLE 2:** Spearman rank correlation coefficients between ceramides and traditional cardiovascular risk factors for the whole study population (n = 695).

	Age	BMI	TC	TG	LDL-C	HDL-C	SdLDL	FFA	Hs-CRP	FBG	HbA1c
Cer14:0	0.065	−0.123**	0.496**	0.268**	0.426**	0.129**	0.385**	0.089*	0.098*	−0.077*	−0.026
Cer16:0	0.036	−0.100**	0.575**	0.362**	0.520**	−0.001	0.481**	0.189**	0.203**	0.086*	0.158**
Cer18:0	−0.007	0.052	0.324**	0.325**	0.242**	−0.061	0.323**	0.296**	0.140**	0.068	0.178**
Cer20:0	−0.020	0.055	0.299**	0.359**	0.247**	−0.130**	0.331**	0.192**	0.138**	0.047	0.156**
Cer22:0	−0.105**	0.033	0.550**	0.453**	0.505**	−0.060	0.572**	0.128**	0.195**	0.084*	0.209**
Cer24:1	−0.009	−0.041	0.402**	0.380**	0.306**	−0.070	0.347**	0.217**	0.169**	0.042	0.114**

*p-value for comparison <0.05, **p-value for comparison <0.01. Non-significant correlation (*p* > 0.05) is marked with NS.

BMI, body mass index; TC, total cholesterol; TG, triglyceride; HDL-C, high-density lipoprotein cholesterol; LDL-C, low-density lipoprotein cholesterol; Hs-CRP, High sensitivity C-reactive protein; FBG, fasting blood glucose; HbA1c, glycated hemoglobin A1c.

### 3.3 Plasma ceramides level in the MS and nonMS groups

**FIGURE 3:**
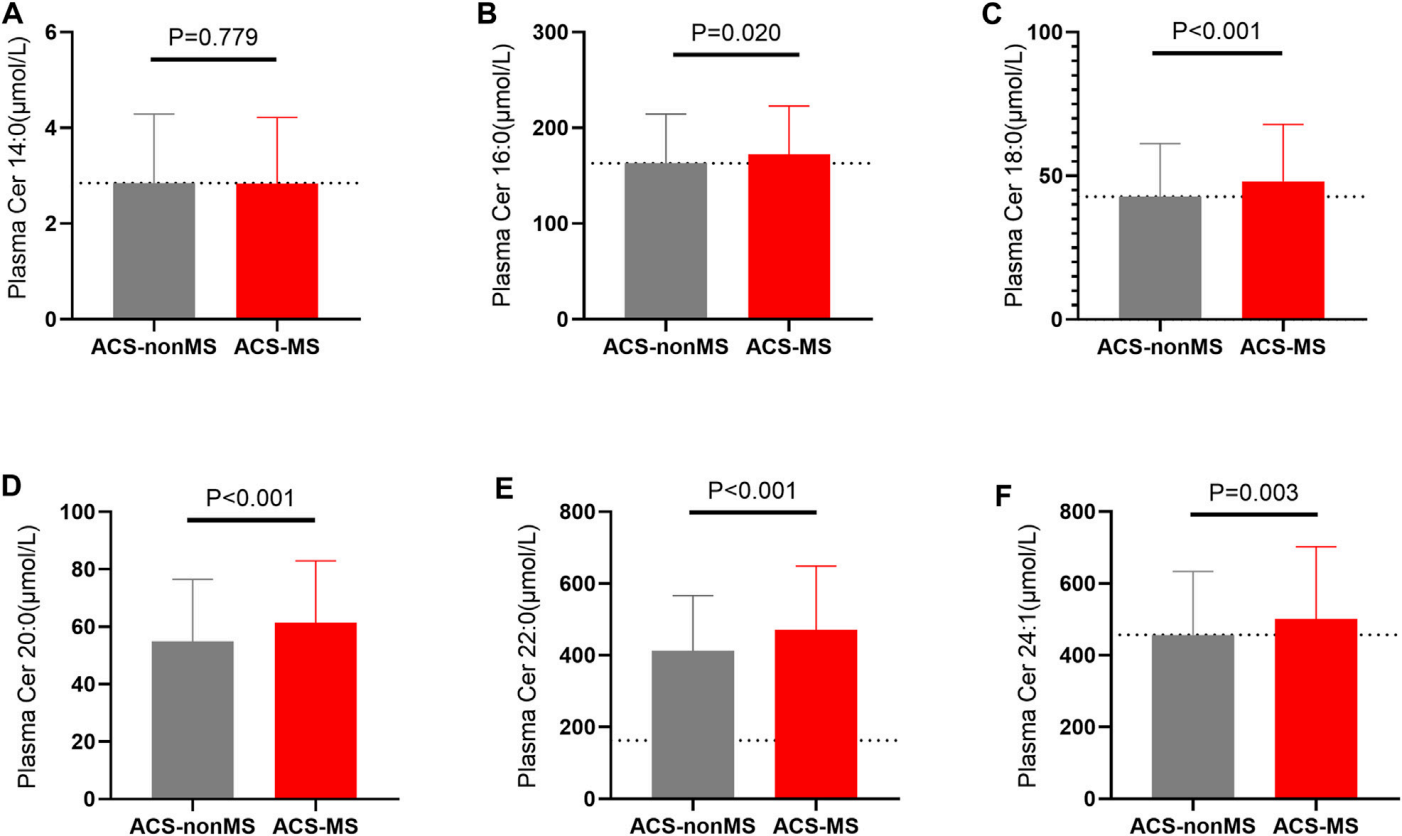
**(A–F)**: A comparison of plasma ceramides levels in patients with only acute coronary syndrome (ACS-nonMS) and those with comorbid acute coronary syndrome and metabolic syndrome (ACS-MS). **(A)** Comparison of Cer14:0 levels between ACS-nonMS patients and (ACS-MS). **(B)** Comparison of Cer16:0 levels between ACS-nonMS patients and (ACS-MS). **(C)** Comparison of Cer18:0 levels between ACS-nonMS patients and (ACS-MS). **(D)** Comparison of Cer20:0 levels between ACS-nonMS patients and (ACS-MS). **(E)** Comparison of Cer22:0 levels between ACS-nonMS patients and (ACS-MS). **(F)** Comparison of Cer24:1 levels between ACS-nonMS patients and (ACS-MS). *p-value for comparison <0.05; **p-value for comparison <0.01; ***p-value for comparison <0.001; ****p-value for comparison <0.0001.

Compared with the ACS-nonMS group, the levels of Cer16:0 (*p* = 0.021), Cer18:0, Cer20:0, Cer22:0(*p*<0.001), and Cer24:1(*p* = 0.002) in the ACS-MS group were higher, whereas the level of Cer14:0 exhibited no significant difference. (Figure 3). Multiple logistic regression analysis revealed that the ceramide levels are significantly related to ACS-MS (Table 3). Results from both model 1 and **model** 2 supported that the relationship between the ceramide level and the ACS-MS group was significant (*p* < 0).

**TABLE 3:** Association between plasma ceramides (per 1 SD) and the risk of ACS-MS.

	Model	OR	95% CI	*p*-value
C14:0 (per 1 SD)	Model 1	1.001	0.857–1.168	0.991
	Model 2	0.983	0.839–1.152	0.835
C16:0 (per 1 SD)	Model 1	1.213	1.041–1.414	0.013
	Model 2	1.196	1.021–1.400	0.026
C18:0 (per 1 SD)	Model 1	1.335	1.143–1.558	0.000
	Model 2	1.304	1.114–1.527	0.001
C20:0 (per 1 SD)	Model 1	1.356	1.161–1.584	0.000
	Model 2	1.335	1.138–1.566	0.000
C22:0 (per 1 SD)	Model 1	1.429	1.218–1.677	0.000
	Model 2	1.405	1.193–1.654	0.000
C24:1 (per 1 SD)	Model 1	1.276	1.095–1.488	0.002
	Model 2	1.221	1.043–1.429	0.013

The table shows the OR, and 95% CI, of the incidence of comorbid acute coronary syndrome and metabolic syndrome (ACS-MS, associated with an increase in plasma ceramides (per 1 SD). The ORs, were estimated from analyses using logistic regression. The model 1 was adjusted for age and sex. The model 2 was further adjusted for eGFR, HCY, uric acid, Hs-CRP; OR, odds ratio; CI, confidence interval; Hs-CRP, High sensitivity C-reactive protein; eGFR, estimated glomerular filtration rate; HCY, homocysteine.

### 3.4 Plasma ceramides level and the number of MS risk factors

**FIGURE 4:**
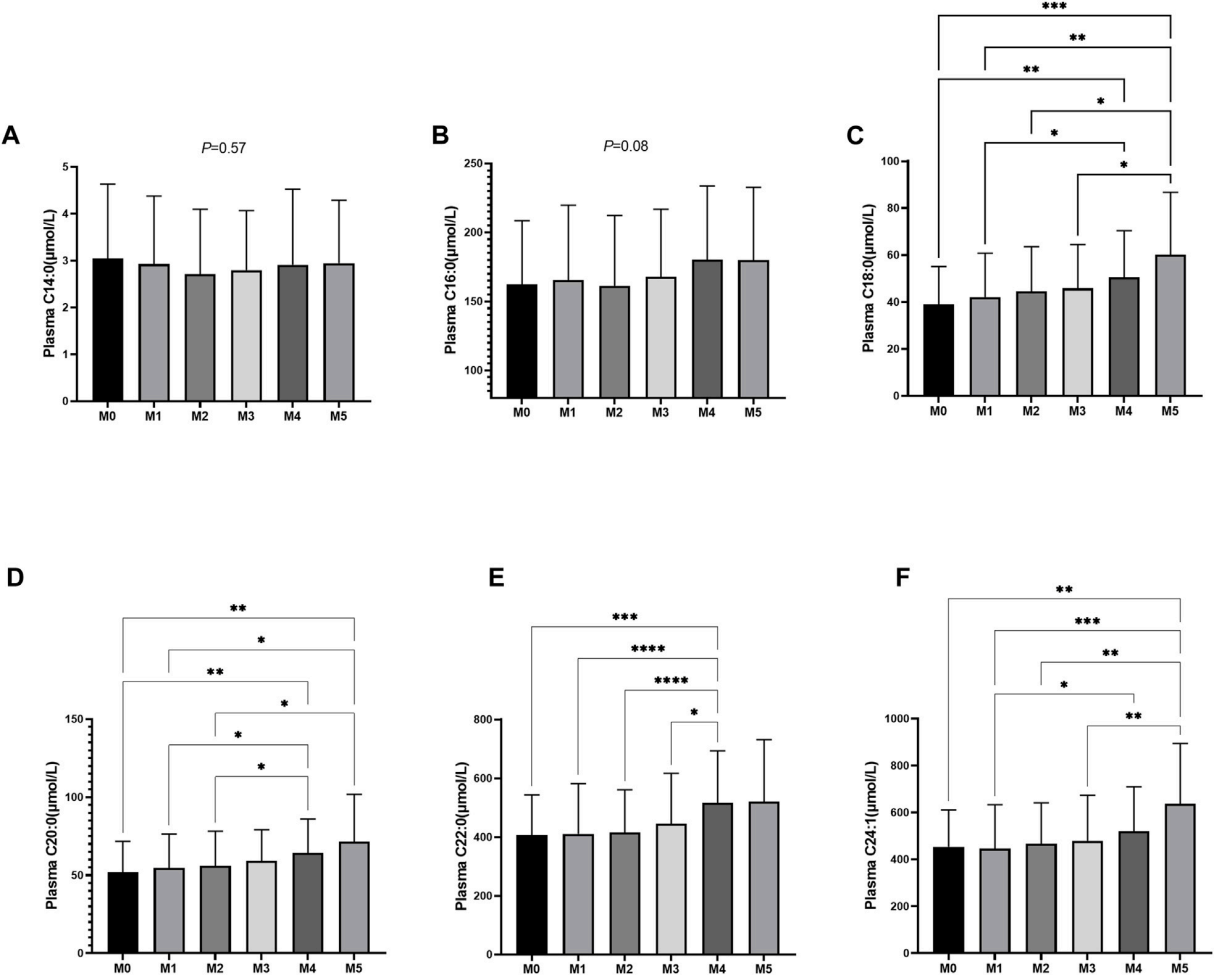
**(A–F)**: Plasma ceramides levels in relation to the number of MS components. **(A)** Comparison of Cer14:0 levels among M0 (*n* = 62), M1 (*n* = 159), M2 (*n* = 188), M3 (*n* = 184), M4 (*n* = 84) and M5 (*n* = 18) subjects. **(B)** Comparison of Cer16:0 levels among M0, M1, M2, M3, M4 and M5 subjects. **(C)** Comparison of Cer18:0 levels among M0, M1, M2, M3, M4 and M5 subjects. **(D)** Comparison of Cer20:0 levels among M0, M1, M2, M3, M4 and M5 subjects. **(E)** Comparison of Cer22:0 levels among M0, M1, M2, M3, M4 and M5 subjects. **(F)** Comparison of Cer24:1 levels among M0, M1, M2, M3, M4 and M5 subjects.*p-value for comparison <0.05; **p-value for comparison <0.01; ***p-value for comparison <0.001; ****p-value for comparison <0.0001.

**FIGURE 5:**
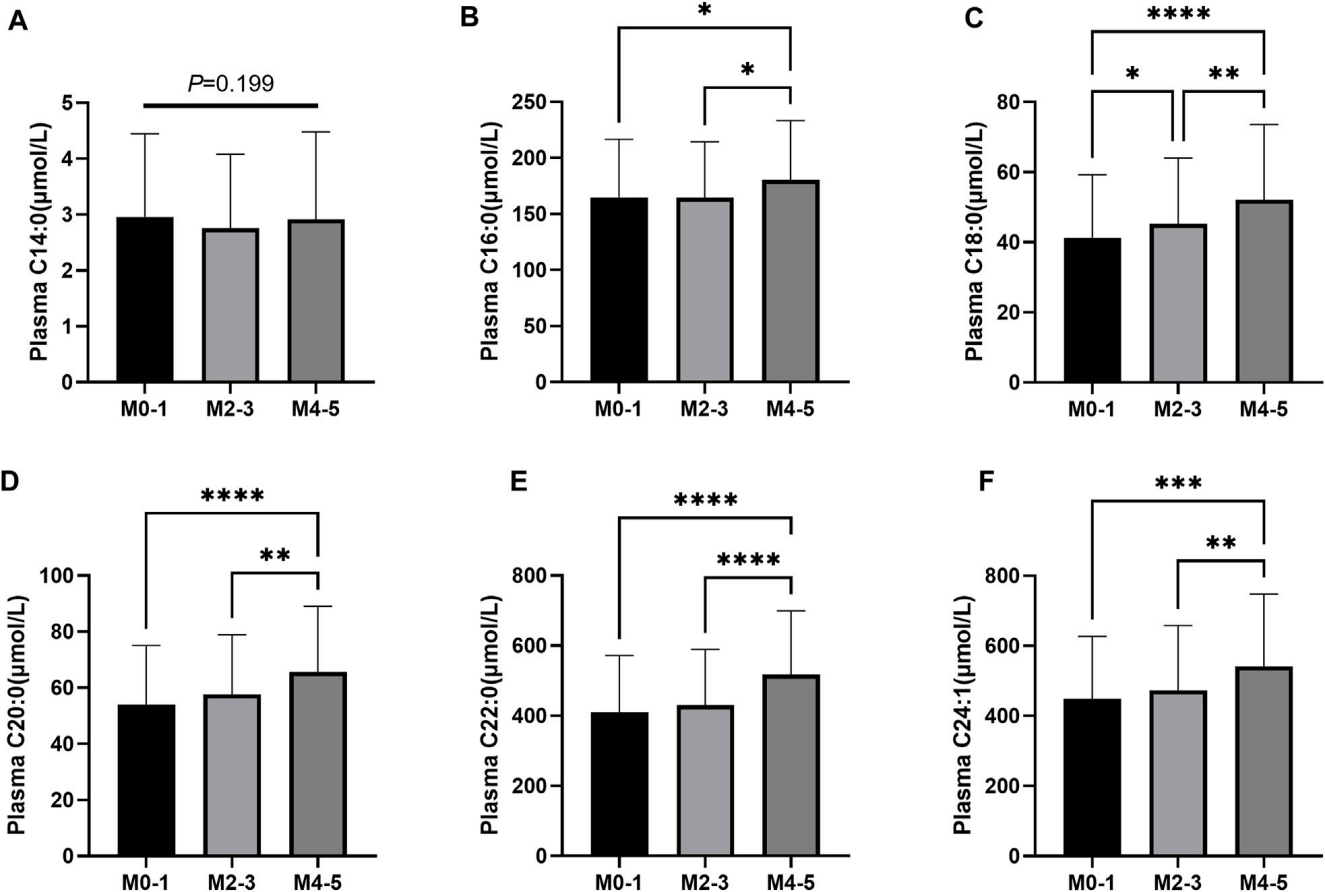
**(A–F)**: Plasma ceramides levels in M0-1, M2-3, M4-5 subjects. **(A)** Comparison of Cer14:0 levels among M0-1 (*n* = 221), M2-3 (*n* = 372), M4-5 (*n* = 102) subjects. **(B)** Comparison of Cer16:0 levels among M0-1, M2-3, M4-5 subjects. **(C)** Comparison of Cer18:0 levels among M0-1, M2-3, M4-5 subjects. **(D)** Comparison of Cer20:0 levels among M0-1, M2-3, M4-5 subjects. **(E)** Comparison of Cer22:0 levels among M0-1, M2-3, M4-5 subjects. **(F)** Comparison of Cer24:1 levels among M0-1, M2-3, M4-5 subjects. *p-value for comparison <0.05; **p-value for comparison <0.01; ***p-value for comparison <0.001; ****p-value for comparison <0.0001.

According to the diagnosed number of MS risk factors, all enrolled patients were divided into the M0, M1, M2, M3, M4, **and** M5 subgroups. The circulating plasma ceramides among those six MS subgroups were assessed and there was no significant difference in C14:0 and C16:0 levels among the subgroups (Figures 4A, B). The levels of Cer18:0, Cer20:0, Cer22:0, and Cer24:1 were significantly elevated with an increasing number of MS risk factors (*p* values ranging from <0.0001 to <0.05 for the general trend) (Figures 4C–F) as labeled by number of asterisks. However, no significant difference of any plasma ceramide levels was observed among subgroups with fewer number of MS risk factors (M0, M1, M2, and M3). We then regrouped patients according to individual MS **risk** factors as described in the earlier session and repeated the same analysis (Figure 5). The plasma Cer18:0, Cer20:0, Cer22:0, **and** Cer24:1 levels were still significantly associated with an increasing number of the MS risk factors (*p* values ranging from <0.0001 to <0.001 for the general trend) while only circulating Cer18:0 level increased progressively from M0-1 to M2-3 subgroup (*p* < 0.05 for the trend).

### 3.5 Correlation between C18:0 and MS risk factors

**FIGURE 6:**
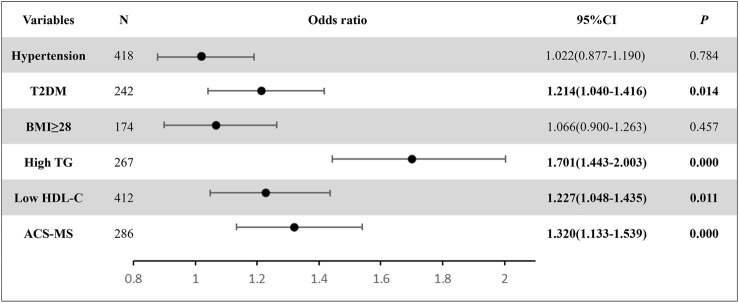
The binary logistic regression results displayed the relationship between Cer18:0 (per 1 SD) and the six ACS risk conditions. The ORs were estimated from analyses using logistic regression. The N value represents the number of diagnosed subjects from the entire study.

The binary logistic regression analysis was performed to further investigate the association between Cer18:0 and MS risk factors. With an increased plasma level of Cer18:0, the risk of MS (*p* < 0.001, OR = 1.320, 95% CI: 1133–1.539), T2DM (*p* = 0.014, OR = 1.214, 95% CI: 1.040–1.416), Low HDL-C (*p* = 0.011 OR = 1.227, 95% CI:1.048–1.435) and High TG (*p* < 0.001 OR = 1.701, 95% CI:1.443–2.003) (Figure 6) also increased.

## 4 Discussion

Acute coronary syndrome (ACS) is a prevalent clinical problem with high cardiovascular risk and mortality. The risk of ACS can significantly increase when the patient is also suffering from metabolic syndrome (MS), which significantly affects the mortality, morbidity, and quality of life of the diagnosed patients (Niedziela et al., 2014; Mak et al., 2021). In this study, we aimed to investigate the relationship between ACS and MS, and to explore the potential use of plasma ceramides as a biomarker for ACS patients. The five major MS risk factors investigated were 1) central obesity: waist circumference (WC) of ≥90 cm in men and ≥85 cm in women. 2) Hypertriglyceridemia (≥1.7 mmol/L), 3) low HDL-C (<1.04 mmol/L), 4) hypertension and 5) Type 2 diabetes. An overview of the clinical characteristics of the 695 enrolled patients categorized by disease status were summarized in Table 1, with 286 subjects in ACS-MS group and 409 in ACS-nonMS group. The demographic and laboratory values of both groups were also summarized, and the data was subjected to the Z-Test. The *p*-values for the conventional cardiovascular risk factors such as BMI, T2DM, hypertension, high triglyceride (High TG), and low HDL-C content were 0.0, suggesting that the occurrence of those phenomena did not take place by chance and the differences between these **characteristics** were significant. A closer look at the *p*-values of these cardiovascular risks supported our hypothesis that patients **who** developed both ACS and MS were more likely to experience a larger chance of ACS morbidity and poorer healthy conditions, including higher BMI, higher likelihood of hypertension, and T2DM, higher plasma triglyceride levels, and lower HLD-C. Additionally, ACS-MS patients also had significantly higher levels of sdLDL, FBG, and uric acid, which contributed to extra risks for ACS patients. The relationship between these cardiovascular risks and metabolic disorders was examined within the ACS-MS group using a differential prevalence study and visualized in a heat map (Figure 2), showing a gradual increase in cardiovascular risks with the accumulation of MS risk factors.

So far, we established that there existed significant differences in the levels of ACS risks between ACS-MS and ACS-nonMS groups and that there was a relationship between the cardiovascular risks and MS risk factors within the ACS-MS group. The purpose of this study was to determine whether plasma ceramides could serve as feasible biomarkers that represent overall metabolic risk. To do so, we quantified the concentrations of six selected plasma ceramides (Cer14:0, Cer16:0, Cer18:0, Cer20:0, Cer22:0, and Cer24:1) in all 695 enrolled patients in both groups using LC-MS/MS assay. The Pearson correlation coefficients for the levels of individual ceramides and traditional cardiovascular risk factors for the entire study population were calculated and tabulated in Table 2. Mild-to-moderate correlations **were** established between six selected ceramides and conventional cardiovascular risk factors, particularly with blood lipids (TC, TG, HDL-C, LDL-C, SdLDL-C, and FFA). We then focused on the relationship between individual ceramides and ACS-MS or ACS-nonMS groups. To begin with, LC-MS/MS measurement results were subjected to the Z-Test for the two groups and the results are summarized in Figure 3. Results from the Z-Test indicated that all ceramides except Cer14:0 and Cer16:0 showed significant differences between the two groups, with *p*-values <0.001 for Cer18:0, Cer20:0, and Cer22:0 and *p* = 0.002 for Cer24:1, which was consistent with the literature findings and supported our hypothesis that plasma ceramide levels increased progressively with increasing numbers of MS risks (Wigger et al., 2017; Anroedh et al., 2018; Neeland et al., 2018). To explore our assumption from a different angle, multiple logistic regression analysis was used to investigate the relationship between individual ceramide and the occurrence of ACS-MS. Results from both model 1 (adjusted for age and sex) and 2 (further adjusted for eGFR, HCY, uric acid, Hs-CRP) (Table 3) showed that the relationship between plasma ceramide levels (Cer18:0, Cer20:0, Cer22:0, and Cer24:1) and ACS-MS group was significant with *p*-values close to 0.000 (95% CI) and that there was a mild relationship between Cer16:0 and ACS-MS group with a *p*-value of 0.013 for model 1 and 0.026 for model 2. The fact that no significant difference was observed between the plasma level of ceramide Cer14:0 or Cer16:0 might have excluded the option of choosing either of them as the potential biomarker for ACS but further research **still** required for confirmation.

Following our initial finding that certain plasma ceramides (Cer18:0, Cer20:0, Cer22:0, and Cer24:1) showed a dysregulated pattern in ACS-MS group, we needed to further **examine** the individual responses of ceramides to MS risk factors. To this end, the whole study population was reclassified into six subgroups (M0, M1, M2, M3, M4, and M5), with M0 representing subjects with only ACS and M5 representing subjects with up to five risk factors. It is worth mentioning that this study only considered the number of MS risk factors, not the specific types of risk factors. The differences in plasma ceramides (Cer14:0, Cer16:0, Cer18:0, Cer20:0, Cer22:0, and Cer24:1) levels between subgroups were analyzed using ANOVA tests. The cut-off *p*-value was 0.05 meaning that any *p*-value larger than 0.05 indicated that there did not exist any significant difference in plasma ceramide concentration between the compared groups. The results, consistent with our previous findings in the ACS-MS and ACS-nonMS groups, showed no significant difference in Cer14:0 and Cer16:0 levels among the subgroups M0 and M5 with a *p*-value of 0.57 for Cer14:0 and 0.08 for Cer16:0 (Figure 4A, B). The study found that plasma levels of Cer18:0, Cer20:0, Cer22:0, and Cer24:1 increased significantly with an increasing number of MS risk factors. Specifically, the *p*-values were <0.001, <0.01, and <0.01 for Cer18:0, Cer20:0, and Cer24:1, respectively. However, there was no significant elevation observed for Cer22:0 between subgroups M0 and M5 (Figure 4E), but a *p*-value of <0.001 was observed when comparing Cer22:0 concentrations between subgroups M0 and M4, confirming the overall trend. When analyzing a smaller number of MS risk factors (i.e., M0, M1, M2, and M3), the trend was less pronounced, and the statistical analysis showed no significant variation in the plasma concentrations of Cer18:0, Cer20:0, Cer22:0, and Cer24:1 across these subgroups. This suggested that the ANOVA test results did not indicate that the plasma levels of Cer18:0, Cer20:0, Cer22:0, and Cer24:1 were sensitive enough to be associated with the presence of only a few MS factors. However, this might have been due to the limited sample size of the study. Nonetheless, as MS risk factors are both cumulative and synergistic, it is common for patients to present with only a few MS risks. Therefore, to explore whether individual plasma ceramide levels respond to specific combinations of MS risk factors involving a smaller number, the study population had to be regrouped.

The 695 enrolled patients were categorized into three groups based on their combination of MS risk factors. For instance, those with 0 and 1 M risk factors were regrouped as M0-1, while those with 2 and 3 M risk factors were regrouped as M2-3, and those with 4 and 5 M risk factors were regrouped as M4-5. By reducing the number of subgroups from six to three, the sample size in each subgroup substantially increased, which could compensate for the limited overall sample size and be advantageous for the statistical analysis. Another ANOVA test was repeated on all six selected plasma ceramide levels across **these** three groups and **the** results **are** summarized in Figure 5. Repeatedly, ceramide Cer14:0 showed **a** similar trend where no significant relationship between its plasma level and **the** number or combination of MS risk factors could be based **on** a *p*-value of 0.199 and thus we could firmly state that it lacked the potentiality of being a blood biomarker for ACS. Among the rest of the selected ceramides, Cer16:0 gave the weakest link between its plasma level and the occurrence of MS risk factors. The plasma Cer18:0, Cer20:0, Cer22:0, **and** Cer24:1 levels were still significantly associated with an increasing number of the MS risk factors (*p* < 0.001 for the trend), making them all ACS biomarkers likely. On top of that, only Cer18:0 showed its potential as the most feasible candidate since its plasma levels increased most sensitively and progressively with **a** minor change in MS risk factor combination with a *p*-value < 0.05 between subgroups M0-1 and M2-3.

To validate the findings from the ANOVA tests, a binary logistic regression analysis was conducted to further examine the link between circulating ceramide Cer18:0 and individual ACS risk factors, as depicted in Figure 6. The study population included individuals diagnosed with six ACS risk conditions, namely, hypertension, T2DM, BMI ≥ 28, high TG, low HDL-C, and ACS-MS. The odds ratio between an increase in plasma Cer18:0 level and the occurrence of each individual risk factor was greater than 1, with a 95% confidence interval. However, the *p*-values associated with hypertension and BMI ≥ 28 were notably higher than the cut-off value of 0.20 (0.784 for hypertension and 0.457 for BMI ≥ 28), whereas the *p*-values related to the other investigated risk factors were lower than the cut-off value (0.014 for T2DM, 0.000 for High TG and ACS-MS, 0.011 for Low HDL-C). The combined odds ratio and *p*-values results indicated that an increase in plasma Cer18:0 level was associated with the likelihood of subjects being diagnosed with ACS with these individual risk factors, but a statistically significant relationship could only be established between Cer18:0 and certain risk factors (T2DM, High TG, Low HDL-C, and ACS-MS). The elevation of plasma Cer18:0 level, coupled with minor changes in certain individual MS risk, suggested that it may serve as a suitable biomarker for identifying patients in their early MS stages and could be valuable in MS risk stratification. This finding is similar to other studies (Hilvo et al., 2020a; Petrocelli et al., 2020; Tu et al., 2020).

There is abundant published data demonstrating that ceramides have a unique utility as an efficient risk stratifier/decision-making tool for cardiovascular disease (CVD) even after adjustment for multiple risk factors. Gui et al. found that plasma levels of three ceramides [Cer(d18:1/16:0), Cer(d18:1/22:0), and Cer(d18:1/24:0)] were significantly higher in acute ischemic stroke patients than in controls and were associated with stroke risk and clinical severity [odds ratios for one IQR increase: 2.15 (1.42–2.99); 2.90 (2.13–4.01), and 1.29 (1.10–1.69), respectively] (Gui et al., 2020). Lemaitre et al. found that Cer(d18:1/16:0) was associated with a higher risk of incident heart failure, while Cer(d18:1/22:0) was associated with a lower risk in a study of 4,249 participants. These findings identify lipidomic biomarkers that may be useful for determining heart failure risk (Lemaitre et al., 2019). The ceramides-based scores (CERT1, CERT2, dScore, and SIC score) using the concentrations of circulating ceramides have been developed and adapted for routine clinical practice (Laaksonen et al., 2016b; Hilvo et al., 2018; Hilvo et al., 2020b; Poss et al., 2020). Additionally, analysis of ceramides or ceramide ratios in subjects with both acute coronary syndrome and diabetes mellitus may provide new options for the evaluation of cardiometabolic disease risk, supported by a wealth of evidence (Chavez and Summers, 2012; Maceyk et al., 2014; Hilvo et al., 2018). Regarding the clinical research area, a recent study involving overweight but healthy monkeys showed a strong correlation between ceramides and the onset of early-stage metabolic syndrome symptoms, highlighting the relevance of circulating ceramides in identifying risk between healthy and impaired study groups. This highlights the potential of ceramides as a simple and minimally invasive diagnostic tool for metabolic syndrome (Smith et al., 2022).

It is important to note that MS risk factors are not only cumulative but also synergistic, meaning that a greater number of MS risk factors or poor control of individual MS risk factors can both result in higher ACS morbidity and mortality. After reviewing our work’s findings, we strongly suggest that plasma ceramide levels, specifically Cer18:0, hold great potential as a biomarker for diagnosing, determining disease stage, and developing personalized treatment plans for ACS-MS events. This holds true even if there are slight changes in MS risk status. Cer18:0 may be used for follow-up and to further motivate patients to initiate or continue medical and life-style recommendations. There is strong evidence that ceramide scores (CERT1 and CERT2) can be used to select CHD patients who have the highest risk and may benefit from more thorough examinations and perhaps more aggressive treatments such as PCSK9 inhibitor or methotrexate. (Ridker, 2016; Sabatine et al., 2018). Another aspect of ceramide testing is its potential to motivate patient adherence, either to medication or lifestyle changes (Hilvo et al., 2018). Obese individuals have higher platelet reactivity than normal weight individuals, possibly due to the production of bioactive substances and hormones by adipose tissue, such as leptin, adiponectin, TNF-α, interleukin-6, and resistin, that can directly or indirectly affect platelet function (Beavers et al., 2015). High levels of platelet aggregation and turnover are also observed in patients with insulin resistance and hyperglycemia (Neergaard-Petersen et al., 2016). Our research suggests that it might be possible to guide the antiplatelet regimen for ACS patients. Thus, ceramide-based identification of ACS patients at high metabolic risk could be beneficial. Nonetheless, it remains to be determined whether combining ceramides in a panel with other lipids or cytokines would be more advantageous and yield higher sensitivity and reliability. Therefore, the utilization of ceramide scores may offer a more accessible approach than the analysis of individual ceramides, and thus has potential for risk stratification in both primary and secondary cardiometabolic prevention (Hilvo et al., 2020a; Choi et al., 2021; Vasile et al., 2021)**.** This is the question we will be addressing in our next research stage. To validate this association and establish distinctive cut-off values in various populations, we will conduct well-designed, large-scale studies in the future.

## 5 Limitation

This study had several limitations that need to be acknowledged. Firstly, the cross-sectional design did not allow for establishing causal relationships between circulating ceramide and metabolic disorders. Secondly, due to the absence of clinical outcome data, it was not possible to evaluate the prognostic implications of ceramides. Thirdly, follow-up ceramide analysis was not conducted, which limited our ability to investigate dynamic changes. In addition, as a single-center study with a relatively small sample size, our findings require confirmation through longitudinal studies with larger sample sizes. Despite these limitations, this study was the first to examine the association between individual plasma ceramides and overall cardiometabolic risk in Chinese individuals, and it provided valuable insights into this critical area of research.

## 6 Conclusion

Our study found a significant link between circulating plasma ceramides and conventional cardiovascular risk factors in ACS patients. These results indicated that Cer18:0 may serve as a valuable surrogate biomarker for metabolic profiles, which can assist in developing preventative treatment strategies for ACS patients. An essential aspect for further research is to determine the predictive value of Cer18:0 for cardiovascular complications in ACS patients with metabolic abnormalities.

## Data Availability

The original contributions presented in the study are included in the article/Supplementary Material, further inquiries can be directed to the corresponding authors.
